# DMOIT: denoised multi-omics integration approach based on transformer multi-head self-attention mechanism

**DOI:** 10.3389/fgene.2024.1488683

**Published:** 2024-12-10

**Authors:** Zhe Liu, Taesung Park

**Affiliations:** ^1^ Interdisciplinary Program in Bioinformatics, Seoul National University, Seoul, Republic of Korea; ^2^ Department of Statistics, Seoul National University, Seoul, Republic of Korea

**Keywords:** multi-omics integration, survival time prediction, deep learning, machine learning, multi-head self-attention

## Abstract

Multi-omics data integration has become increasingly crucial for a deeper understanding of the complexity of biological systems. However, effectively integrating and analyzing multi-omics data remains challenging due to their heterogeneity and high dimensionality. Existing methods often struggle with noise, redundant features, and the complex interactions between different omics layers, leading to suboptimal performance. Additionally, they face difficulties in adequately capturing intra-omics interactions due to simplistic concatenation techiniques, and they risk losing critical inter-omics interaction information when using hierarchical attention layers. To address these challenges, we propose a novel Denoised Multi-Omics Integration approach that leverages the Transformer multi-head self-attention mechanism (DMOIT). DMOIT consists of three key modules: a generative adversarial imputation network for handling missing values, a sampling-based robust feature selection module to reduce noise and redundant features, and a multi-head self-attention (MHSA) based feature extractor with a noval architecture that enchance the intra-omics interaction capture. We validated model porformance using cancer datasets from the Cancer Genome Atlas (TCGA), conducting two tasks: survival time classification across different cancer types and estrogen receptor status classification for breast cancer. Our results show that DMOIT outperforms traditional machine learning methods and the state-of-the-art integration method MoGCN in terms of accuracy and weighted F1 score. Furthermore, we compared DMOIT with various alternative MHSA-based architectures to further validate our approach. Our results show that DMOIT consistently outperforms these models across various cancer types and different omics combinations. The strong performance and robustness of DMOIT demonstrate its potential as a valuable tool for integrating multi-omics data across various applications.

## 1 Introduction

With the advent of high-throughput sequencing technologies, various types of omics data, including genomics, transcriptomics, and proteomics data, have become increasingly accessible. The pathogenesis of diseases often involves complex interactions across multiple biological levels and factors. Consequently, single-omics data provide only partial insights into biological processes, often failing to capture other critical factors and leading to an incomplete understanding of disease mechanisms. In contrast, multi-omics approaches offer the potential to reveal new biological insights that are not apparent when single-omics data are used alone (Yan et al., 2018). Therefore, the integration of multiple omics data is essential for achieving a comprehensive and complementary understanding of complex disease occurrence and progression, thereby further advancing personalized medicine (Hasin et al., 2017). However, integrating multi-omics data presents several challenges. Firstly, there is significant heterogeneity among different omics data types (genomics, transcriptomics, and proteomics), making integration complex due to varying data formats and scales (López de Maturana et al., 2019). Additionally, missing values and noise in the data can impact accuracy (Flores et al., 2023), while the large scale of multi-omics datasets demands substantial computational resources and efficient algorithms (Fondi and Liò, 2015). Lastly, combining information across different biological levels adds another layer of complexity, and interpreting and visualizing the integrated results can be challenging (Krassowski et al., 2020).

In the past decade, researchers have made significant progress in developing tools for multi-omics data integration. Dimension reduction-based methods have been foundational in multi-omics data integration. For example, canonical correlation analysis (Qi et al., 2021) is commonly used to evaluate the correlation between feature sets in different omics data. principal component analysis transforms relevant variables into linearly uncorrelated principal components through orthogonal transformation. However, those approaches typically assume linear relationships between features and fail to capture nonlinear relationships. Deep learning-based models can better capture complex nonlinear relationships due to multi-level neural network architecture, making them crucial tools in multi-omics data integration (He et al., 2023). Convolutional neural networks and Recurrent neural networks are utilized to handle high-dimensional and nonlinear multi-omics data, extracting complex features from them (Kang et al., 2022). In addition, encoder-decoder models, such as variational autoencoder (Hira et al., 2021) and generative adversarial network (Ahmed et al., 2022) are widely used for the integration and generation of multi-omics data, achieving dimensionality reduction by obtaining intermediate latent feature representations. Graph convolutional networks (Li et al., 2022) facilitate efficient information dissemination and aggregation by modeling the complex relationships and graph structure characteristics between data and are also applied to multi-omics data integration.

In recent years, the attention mechanism has become a hot topic in deep learning-based integration methods. Researchers (Gong et al., 2023) have applied attention mechanisms to reduce dimensionality and learn feature representations for each omics data type. Another study (Pang et al., 2023) introduced a hierarchical attention layer based on the biological central dogma to enhance data integration effectiveness. These methods demonstrate the enormous potential of attention mechanisms in multi-omics data integration. Additionally, some researchers (Zhang et al., 2022) constructed a Graph attention network and group-level attention mechanism to learn embedding representations. However, existing attention-based methods often face limitations. Some approaches (Gong et al., 2023) typically simply input each omics data into a separate attention layer, focusing only on the intra-omics interactions and ignoring the inter-omics interaction. Other approaches concatenate multiple omics datasets before applying a single attention layer (Wang et al., 2024; Pan et al., 2023). Given the heterogeneity of omics data, this approach may not effectively attend to each data type, making it challenging to capture intra-omics integrations. Additionally, due to the high dimensionality of omics data, single-head self-attention mechanisms might be inadequate in capturing subtle interactions. Overall, these methods may not fully exploit the potential value in multi-omics data.

Effective data preprocessing is also critical for optimizing performance in multi-omics integration frameworks. The characteristics of high-dimensional omics data, such as missing values and significant noise, make multi-omics data integration challenging. It has been proven that missing values in high-dimensional omics data can adversely affect downstream analyses (Flores et al., 2023). Therefore, addressing missing values is essential for maintain data quality. However, existing attention-based multi-omics integration methods often rely on simplistic imputation strategies such as zero, mean, or median imputation. These methods often fail to account for the complex correlations within omics data, potentially introducing unnecessary noise from imputed values. Moreover, discarding features with missing values might result in losing important information. Additionally, high-dimensional data also often contain numerous redundant features that may be selected by chance and degrade performance. Therefore, feature selection is a vital preprocessing step aimed at reducing noisy features and effectively decreasing dimensionality. While many integration frameworks select features based solely on the highest variance, which can lead to the inclusion of noisy and unstable features due to noise, outliers, and data disturbances.

**FIGURE 1:**
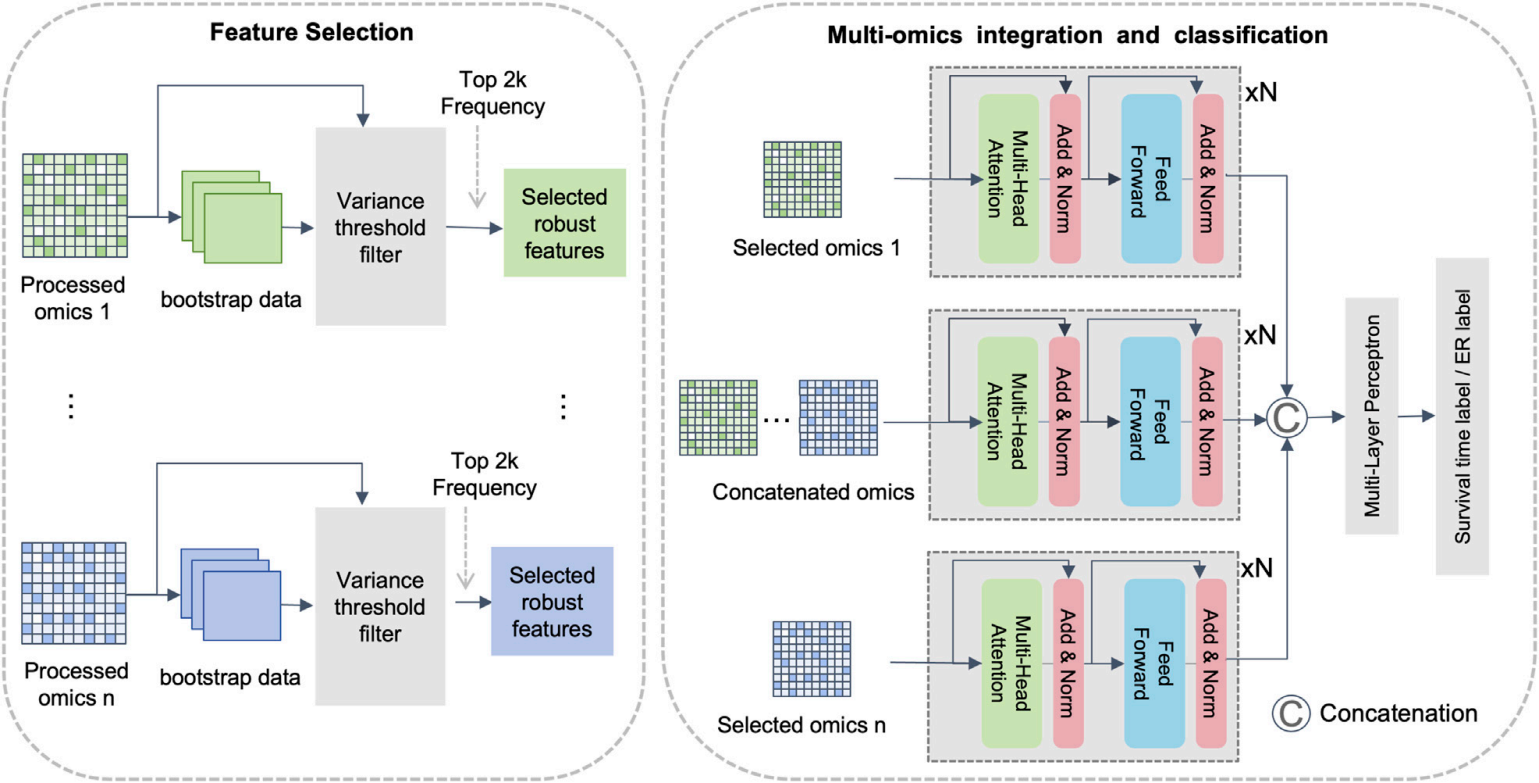
Overview of the proposed multi-omics integration framework. Note: This framework is primarily divided into three modules. The first module utilizes the GAIN model for imputation, the second module is for feature selection based on bootstrap sampling, and the third module is an integration module based on multi-head self-attention mechanism.

To improve feature relevance, reduce noise, and capture both intra- and inter-omics interactions, we propose DMOIT, a novel denoised multi-omics integration approach. As shown in Figure 1, DMOIT includes three main modules. First, the Generative Adversarial Imputation Network (GAIN) (Yoon et al., 2018) module is introduced to learn feature distributions and impute missing values. Second, the Robust Feature Selection (RFS) module, based on bootstrap sampling, is employed to identify a denoised and stable feature set. Finally, a feature extractor leveraging the transformer multi-head self-attention (MHSA) mechanism is constructed to integrate multi-omics data, capturing both intra- and inter-omics interactions. Empirical studies across various cancer types and omics combinations demonstrate that DMOIT significantly enhances performance in multi-omics data integration and analysis.

## 2 Materials and methods

### 2.1 Data acquisition and preprocessing

We evaluated our proposed framework using three types of omics data: mRNA expression profiles, DNA methylation (Met), and copy number variation (CNV). The datasets were obtained from the UCSC Xena web browser, a resource that includes multi-omics and clinical data of cancer patients from The Cancer Genome Atlas (TCGA) project. We filtered samples with complete data for all three types of omics and clinical information. To enhance the reliability of our results, we focused on the top four TCGA cancer types with the most samples after filtering, including breast invasive carcinoma (BRCA), head and neck squamous cell carcinoma (HNSC), liver hepatocellular carcinoma (LIHC), and stomach adenocarcinoma (STAD). To validate the effectiveness and robustness of our proposed model, we applied DMOIT to survival time classification tasks across these four cancer types and the estrogen receptor (ER) status classification task for BRCA. In our survival time classification task, rather than using the median survival time, we set the threshold to the nearest integer to the median to better align with practical clinical applications. Patients with survival times greater than the threshold are labeled as long-term survivors (LTS), while those with shorter survival times are labeled as non-long-term survivors (non-LTS). The distribution of survival time labels for the specific cancer types shown in Table 1. For the estrogen receptor (ER) status classification task, we obtained the clinical information from cBioPortal (Gao et al., 2013), categorizing 199 patients as ER positive (ER+) and 55 as ER negative (ER-). During data preprocessing, we first removed features with high missing value rates. Specifically, CNV data was not included in the imputation process because they did not contain any missing data. For the mRNA and Met data, we removed features with 100% missing value rate and then applied min-max scaling to mitigate the impact of magnitude differences between features and ensure that features contribute equally during variance filtering and enhances the performance of subsequent machine learning models. For CNV data, we marked the variations into three types: no (0), decreased copy number (−1), and increased copy number (Yan et al., 2018). A bootstrap sampling-based feature selection module was applied to filter the denoised feature set. A detailed explanation follows in the subsequent sections. We then imputed the mRNA and Met data using the GAIN model. Details of data preprocessing are provided in Supplementary Figures S1, S2.

**TABLE 1:** Sample distributions of long-term survivors (LTS) and non-long-term survivors (non-LTS) across the four cancer types.

Dataset	Total samples	LTS	Non - LTS
BRCA	783	416	367
HNSC	514	370	144
LIHC	368	251	117
STAD	366	223	143

### 2.2 Robust feature selection module

High-dimensional data often exhibits characteristics such as noise and redundant features. Noise can introduce random variations that obscure the true signal in the data, while redundant features can lead to overfitting and increased computational complexity. These issues may further result in unstable feature selection outcomes and degrade model performance. Traditionally, researchers integrating multi-omics data have relied on a single variance filter for feature pre-selection. However, including noisy samples—especially those with extreme values due to errors in sequencing technology or data entry—can skew the results during the single variance filtering step. This can lead to an overemphasis on certain features, causing the selection of unreliable or misleading data. To address this, we incorporated a robust feature selection (RFS) module that assesses features stability using the bootstrapping resampling technique, a widely recognized method for evaluating feature reliability. Our approach was inspired by the work of Cho et al. (2010), who employed a bootstrap method to select stable feature sets and proposed a new measure called Bootstrap Selection Stability. Furthermore, previous studies have demonstrated that the feature selection results can be significantly influenced by data disturbances; even minor alterations in the sample data can lead to the substantial changes in outcomes. To address this issue, Pes (2020) also proposed employing bootstrap sampling to conduct multiple feature selections, subsequently identifying a stable dataset based on the frequency of selection. This highlights the necessity for robust feature selection methods, especially in multi-omics analyses. In our RFS module, we generated ten bootstrap samples for each type of omics data. Bootstrap sampling involves creating multiple subsets of the original data via random sampling with replacement. Bootstrap sampling simulates data variability and helps to assess the consistency of feature importance under different perturbations. For each bootstrap sample, we filtered the features with the highest variance, as these are typically more informative. We then selected the top features based on their frequency of selection across all bootstrap samples. The RFS module ensures that only consistently selected features are chosen, thus enhancing the relevance and robustness of the final feature set.

### 2.3 Generative adversarial imputation network in multi-omics integration

Previous studies (Gunady et al., 2019; Xu et al., 2020; Wang et al., 2023) have shown that generative adversarial network (GAN)-based methods achieve promising results in imputing mRNA expression data. However, the stability of GANs in multi-omics data integration has not been extensively investigated. Given GANs’ ability to learn and mimic any data distribution (Yoon et al., 2018), we hypothesized that they could handle multi-omics data imputation effectively, mitigating noise from missing values. GANs work by training two networks simultaneously: a generator that generates realistic data, and a discriminator that distinguishes between real and synthetic samples. This adversarial training process enables GANs to learn the underlying data distribution accurately and generate realistic data samples that closely align with the true data, thereby reducing noise and improving imputation quality. To avoid introducing additional noise from imputing true observations, we imputed only the missing values in the original data after completing the data generation process. In our study, we imputed mRNA and MET data based on the learned distribution. CNV data was not included in the imputation process because they did not contain any missing data.

### 2.4 Multi-head self-attention based multi-omics data integration

Self-attention allows the model to weigh the importance of different features within the same omics dataset. This capability is crucial for capturing the intricate dependencies and interactions between features both within individual layers and across different omics layers. The self-attention mechanism is mathematically described as follows (Vaswani et al., 2017):

$$\text{Attention}\left(Q,K,V\right)=\text{softmax}\left(\frac{Q\;K^{T}}{\sqrt{d_{k}}}\right)V$$

where Q (query), K (key), and V (value) are matrices derived from the same set of omics features, and where $d_{k\;}$ represents the dimensionality of the keys. This mechanism enables the model to focus on different features and determine which features are most relevant to each other. Expanding on self-attention, the multi-head self-attention (MHSA) mechanism incorporates multiple attention heads to capture a variety of relationships among features. The MHSA mechanism is described as follows:

$$\text{MultiHead}\left(Q,K,V\right)=\text{Concat}\left({\text{head}}_{1},{\text{head}}_{2\;},\ldots ,{\text{head}}_{h}\right)W^{o}$$

where each head is calculated as:

$${\text{head}}_{i}=\text{Attention}\left(QW_{i}^{Q},KW_{i}^{K},VW_{i}^{V}\right)$$

Here, $W_{i}^{Q},W_{i}^{K}$ , and $W_{i}^{V}$ are learned weight projection matrices for the i -th head, and $W^{o}$ is a weight matrix applied to the concatenated outputs of all heads. In the DMOIT framework, we design an MHSA mechanism-based feature extractor with a novel architecture to effectively integrate multi-omics data. Compared with the single-head self-attention mechanism, the MHSA can effectively capture features from various perspectives and subspaces by processing the input data through multiple attention heads, thereby enhancing the model’s ability to detect complex interactions and improving its robustness and stability. Additionally, the MHSA enables parallel computation, significantly increasing efficiency and providing notable advantages, particularly for large-scale multi-omics datasets. Our architecture leverages the MSHA mechanism to capture both intra- and inter-omics integrations effectively. Specifically, each omics dataset is input into separate encoders to fully learn the intra-omics interactions, reducing noise and improving the signal quality. Simultaneously, the concatenated omics data are fed into a shared MHSA-based encoder to capture the inter-omics interactions. This approach ensures that interactions between different omics types are preserved and effectively learned without losing any information. The outputs from the individual and shared encoders are then combined and passed into a multilayer perceptron (MLP) for final prediction. This dual architecture ensures comprehensive learning of both intra- and inter-omics interactions, providing a thorough analysis of individual omics data while maintaining the integrity of inter-omics interactions.

### 2.5 Comparative multi-omics data integration methods

**FIGURE 2:**
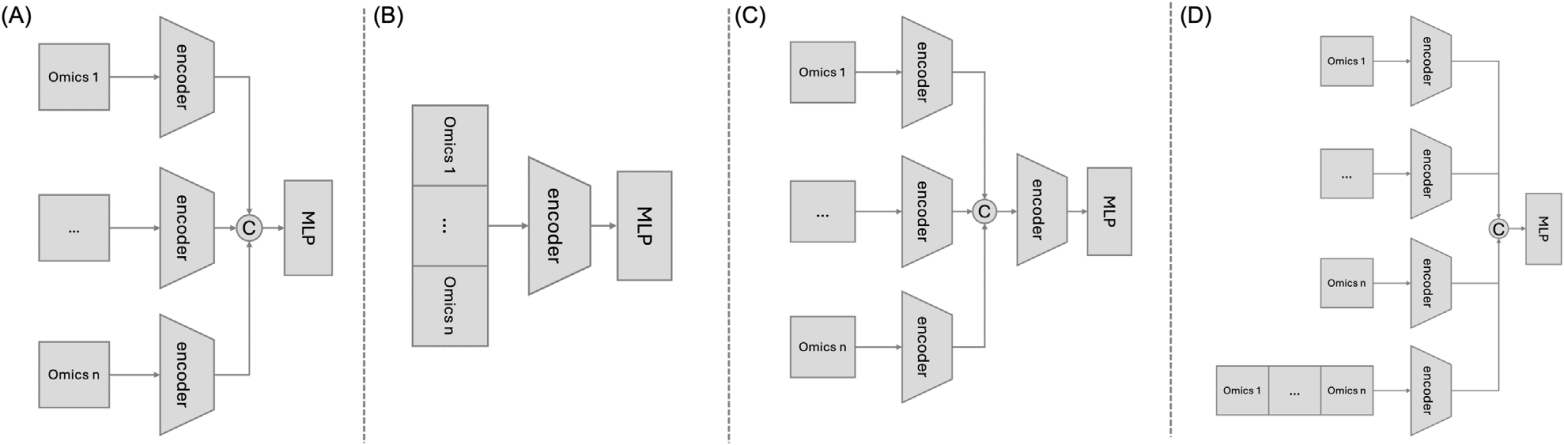
Multi-Omics data integration methods based on multi-head self-attention mechanism. Note: This figure shows four different frameworks based on the multi-head self-attention mechanism. **(A)** M1: Each omics dataset is independently input into its corresponding encoder. **(B)** M2: Multiple omics data are concatenated and then input into a single encoder. **(C)** M3: Each omics dataset is independently input into its corresponding encoder, and the concatenated feature representations are then input into the next layer’s encoder. **(D)** Our proposed approach, DMOIT.

We employed four traditional machine learning models—logistic regression (LR), random forest (RF), support vector machine (SVM), and extreme gradient boosting (XGBoost)-as baseline models, handling multiple omics datasets by concatenating the datasets. Additionally, we included a state-of-the-art method MoGCN. To validate our proposed architecture, we compared it with three alternative MHSA-based feature extractors with different architectures (Figure 2). In these frameworks, omics data is first input into a linear layer before entering the multi-layer attention layers to enhance its representation.

• Model 1 (M1): This model inputs each omics dataset separately into an MHSA layer to capture intra-omics interactions. The outputs from each encoder are then concatenated and passed through an MLP for classification. While M1 excels at capturing intra-omics interactions, it does not address inter-omics interactions.
• Model 2 (M2): Here, multiple omics datasets are concatenated before being input into a single MHSA layer. M2 effectively learns inter-omics interactions and maintains information completeness. However, due to varying noise levels and heterogeneity among omics datasets, it may struggle to adequately attend to each type of omics data, making it challenging to capture intra-omics interactions.
• Model 3 (M3): This model processes each omics dataset with separate MHSA layers to learn intra-omics interactions. The outputs from these layers are then concatenated and fed into an additional MHSA layer to further capture inter-omics interactions. While M3 aims to capture both intra- and inter-omics interactions, there is a risk of losing some inter-omics interaction information during the initial independent processing.

### 2.6 Evaluation methods

We designed two classification tasks to validate each module in our proposed framework: a survival time classification across four cancer types and an ER status classification for breast cancer. Both tasks used 5-fold cross-validation to ensure the robustness of our results. Model performance was evaluated using the mean accuracy and weighted F1-score metrics from the cross-validation.

### 2.7 Training of the DMOIT and other MHSA-based comparison models

The MHSA-based models were developed using PyTorch (version 2.1.2) and scikit-learn. We trained the model for 50 epochs and used a grid search to identify the optimal parameters, utilizing the Adam optimizer for training. To balance performance and computational efficiency, the encoders in each model share identical parameters. The grid parameter combinations are detailed in Table 2.

**TABLE 2:** Hyperparameter settings for grid search.

Hyperparameter	Possible values
learn_rate	[0.001, 0.01]
batch_size	[32, 64, 128]
num_heads	[2, 4, 8]
num_blocks	[1, 2, 3]
dropout_rate	[0.01, 0.1]
dense_dim	[32, 64, 128]

## 3 Results

### 3.1 Evaluation of the GAIN imputation method

Omics data are typically high-dimensional and often contain a large proportion of missing values. In the dataset we downloaded from UCSC Xena, these missing values were treated as zeros. However, it remains uncertain whether these zeros are biologically meaningful or the result of technical issues during sequencing. In this study, we compared the impact of non-imputed data (where missing values were retained as zeros) versus data imputed using various imputation methods on survival time classification tasks. We applied these methods to imputed mRNA and Met omics data from four cancer types, using the XGBoost classifier due to its superior performance among all the traditional machine learning models we tested and considering the computational demands of deep learning approaches. Traditional imputation methods, such as mean, median, and K-nearest neighbor (KNN) (Pujianto et al., 2019), commonly used in previous studies, fail to distinguish between biological zeros and technical zeros, treating them uniformly. In contrast, GAN-based imputation is more likely to preserve biologically meaningful zeros by learning the underlying feature distribution, which in turn enhances the performance of downstream analyses. As shown in Table 3, the mean accuracy and weighted F1 score from 5-fold cross-validation on the test set, as well as the overall averages for each omics dataset and across the four cancer types, demonstrate the effectiveness of different imputation methods. Among the methods tested, the GAN-based GAIN module achieved the highest average testing accuracy of 0.655 and average weighted F1 score of 0.614, outperforming KNN imputation (accuracy: 0.640, F1: 0.600), mean imputation (accuracy: 0.641, F1: 0.596), median (accuracy: 0.648, F1: 0.606), and zero imputation (accuracy: 0.641, F1: 0.596). These findings also suggest that GANs have potential for generalizing to other omics types and highlight their promise for robust and reliable imputation across diverse omics datasets.

**TABLE 3:** Performance of different imputation methods.

Dataset	Method	Accuracy	Weighted F1
BRCA	zero	0.622	0.621
	median	0.623	0.622
	mean	0.622	0.621
	KNN	0.617	0.614
	GAIN	**0.633**	**0.633**
HNSC	zero	0.704	0.612
	median	0.708	0.61
	mean	0.704	0.612
	KNN	0.696	0.609
	GAIN	**0.708**	**0.623**
LIHC	zero	0.641	0.596
	median	0.666	0.627
	mean	0.641	0.596
	KNN	**0.671**	**0.632**
	GAIN	**0.671**	**0.632**
STAD	zero	0.596	0.555
	median	0.596	0.566
	mean	0.596	0.555
	KNN	0.577	0.546
	GAIN	**0.607**	**0.567**
Mean across 4 cancer types	zero	0.641	0.596
	median	0.648	0.606
	mean	0.641	0.596
	KNN	0.640	0.600
	GAIN	**0.655**	**0.614**

Note: The accuracy and weighted F1 score are the averages from 5-fold cross-validation of each cancer type and across four different cancer types. The bold values represent the highest accuracy/F1 score for downstream classification tasks achieved across all datasets imputed by different imputation methods for the current cancer type.

### 3.2 Evaluation of the bootstrap-based robust feature selection module

To evaluate the effectiveness of robust feature selection (RFS) module within the DMOIT framework, we compared it with a direct feature selection method that identifies the top features without bootstrap sampling. Both approaches were assessed through survival time classification tasks with the XGBoost classifier across four cancer types. As shown in Table 4, the RFS module consistently outperformed the direct selection method. For instance, in STAD, accuracy increased from 0.569 to 0.601 and the weighted F1 score rose from 0.534 to 0.559. In LUSC, accuracy improved from 0.686 to 0.694, with the weighted F1 score going up from 0.593 to 0.595. Similar improvements were observed in LIHC, HNSC, and BRCA, with notable gains in both accuracy and F1 scores. These results demonstrate that the RFS module enhances feature selection by effectively handling noise and selecting a stable feature set. It mitigates the impact of data distribution imbalance and dataset-specific sensitivities in high-dimensional data, leading to improved feature relevance and overall performance.

**TABLE 4:** Performance comparison of different feature selection methods.

Dataset	Accuracy		Weighted F1	
	RFS	Direct	RFS	Direct
STAD	**0.601**	0.569	**0.559**	0.534
LIHC	**0.666**	0.649	**0.622**	0.61
HNSC	**0.714**	0.702	**0.631**	0.607
BRCA	**0.627**	0.609	**0.626**	0.608

Note: “Direct” denotes direct filtering of the features based on variance, and “RFS” denotes the bootstrap-based robust feature selection module. The accuracy and weighted F1 score are the averages of 5-fold cross validation in the survival time classification task using XGBoost. The bold values represent the highest accuracy/F1 score between the two feature selection methods for the current cancer type.

### 3.3 Performance of DMOIT under different omics combinations

To evaluate the effectiveness and stability of DMOIT in learning intra- and inter-omics interactions, we compared it against four traditional machine learning baseline models, the state-of-the-art MoGCN method, and three alternative MHSA-based architectures, as detailed in the methods section. This evaluation was conducted on both survival time classification and ER status classification tasks. We tested the generalization ability of these models by comparing their performance across different omics combinations in various cancer types, including single-omics data, paired combinations, and an integrated dataset comprising all three omics types.

To validate how direct concatenation of multiple omics datasets increases data heterogeneity, we first investigated the changes in data properties before and after concatenation. Different features may exhibit distinct data types; for instance, in our study, mRNA and MET are continuous variables, while CNV is a discrete variable. Additionally, distributional differences among omics of the same data type may persist, indicating that heterogeneity is likely to increase after concatenation, as illustrated in the histograms of mean expression levels, coefficient of variation, and Shannon entropy shown in Supplementary Figures S4–S6. This increased heterogeneity may hinder the attention mechanism’s ability to fully capture intra-omics interactions within a specific omics dataset. We further demonstrated the complexity of omics data, particularly the hierarchical clustering and nonlinear relationships among variables, which highlights the need for multi-head attention mechanisms to learn the complex relationships among omics features compared to statistical models and traditional machine learning models. This is illustrated by the correlation heatmap of BRCA mRNA omics in Supplementary Figure S7, as well as the LOESS curve and polynomial fitting for the top 5 mRNA biomarkers in Supplementary Figures S8, S9. These factors present significant challenges for multi-omics integration. Unlike simple concatenation, DMOIT effectively addresses these issues using a multi-head attention mechanism. By learning from each omics dataset individually, we reduce the impact of heterogeneity on intra-omics interactions. Meanwhile, the learning from concatenated data ensures the completeness of inter-omics interactions.

In the survival time classification task (Table 5), DMOIT achieved the highest accuracy and the weighted F1 score across all the omics data combinations in HNSC and LIHC and performed better in at least two out of four omics combinations in BRCA and STAD. For the ER status classification task using all three omics types (Table 6), DMOIT achieved the highest weighted F1 score of 0.937. Although its accuracy was slightly lower than M3, it remains a more efficient choice due to lower computational complexity. Additionally, the original ER dataset exhibited class imbalance; therefore, we conducted a simple experiment to test the impact of varying degrees of data imbalance on DMOIT. We created scenarios with ER positive-to-negative ratios of approximately 1:1, 2:1, and 3:1 by randomly sampling from the larger set of ER-positive cases. As shown in Table 7, DMOIT performed similarly across different levels of imbalance, with accuracy and weighted F1 scores as follows: for the 1:1 ratio, accuracy was 0.927 and the weighted F1 score was 0.927; for the 2:1 ratio, accuracy was 0.933 and the weighted F1 score was 0.932; and for the 3:1 ratio, accuracy was 0.914 and the weighted F1 score was 0.910. Our findings reveal that MHSA-based models, particularly DMOIT, consistently outperform both traditional models and MoGCN. Among the four MHSA-based models, DMOIT consistently exhibits superior performance across various cancer datasets and omics combinations in most scenarios on different tasks, highlighting its effectiveness and robustness in managing complex intra- and inter-omics interactions. Furthermore, our results indicate that mRNA provides the most informative data among single-omics datasets and incorporating a third omics type does not necessarily enhance performance.

**TABLE 5:** Performance of various machine learning models on multi-omics data for different cancer types in survival time classification task.

Cancer type	Omics data	Accuracy								
		LR	RF	SVM	XGB	M1	M2	M3	DMOIT	MoGCN
BRCA	mRNA	0.586	0.595	0.613	0.618	**0.670**				—
	MET	0.544	0.561	0.531	0.598	**0.618**				—
	CNV	0.498	0.503	0.494	0.516	**0.595**				—
	mRNA + MET	0.590	0.586	0.582	0.633	0.667	0.667	**0.669**	**0.669**	0.530
	mRNA + CNV	0.561	0.602	0.527	0.614	0.650	0.641	0.651	**0.655**	0.512
	MET + CNV	0.535	0.542	0.493	0.593	0.605	**0.613**	0.612	0.607	0.506
	mRNA + MET + CNV	0.563	0.604	0.516	0.627	0.662	0.649	**0.669**	0.653	0.525
HNSC	mRNA	0.652	0.704	0.718	0.691	**0.728**				—
	MET	0.665	0.712	0.720	0.702	**0.730**				—
	CNV	0.615	0.691	0.712	0.687	**0.730**				—
	mRNA + MET	0.667	0.720	0.720	0.708	0.728	0.730	**0.732**	**0.732**	0.510
	mRNA + CNV	0.628	0.708	0.716	0.696	0.734	0.734	0.739	**0.741**	0.553
	MET + CNV	0.650	0.710	0.720	0.706	0.737	0.737	0.737	**0.739**	0.531
	mRNA + MET + CNV	0.636	0.716	0.720	0.714	0.737	0.736	0.739	**0.740**	0.533
LIHC	mRNA	0.650	0.682	0.685	0.669	**0.755**				—
	MET	0.628	0.671	0.679	0.660	**0.739**				—
	CNV	0.611	0.636	0.682	0.639	**0.717**				—
	mRNA + MET	0.633	0.682	0.679	0.671	0.755	0.745	0.750	**0.763**	0.658
	mRNA + CNV	0.622	0.685	0.679	0.674	0.734	0.728	0.734	**0.742**	0.669
	MET + CNV	0.650	0.668	0.674	0.655	0.731	0.712	0.725	**0.739**	0.663
	mRNA + MET + CNV	0.644	0.685	0.677	0.666	0.747	0.731	0.736	**0.748**	0.649
STAD	mRNA	0.555	0.623	0.601	0.590	**0.670**				—
	MET	0.481	0.582	0.609	0.558	**0.642**				—
	CNV	0.530	0.538	0.596	0.538	**0.650**				—
	mRNA + MET	0.506	0.590	0.604	0.607	0.677	0.675	0.672	**0.678**	0.577
	mRNA + CNV	0.511	0.607	0.598	0.577	0.642	0.645	0.647	**0.648**	0.585
	MET + CNV	0.462	0.555	0.607	0.536	0.642	**0.656**	0.645	0.642	0.609
	mRNA + MET + CNV	0.503	0.612	0.601	0.601	0.650	0.649	0.650	**0.651**	0.563

Cancer type	Omics data	Weighted F1-Score								
		LR	RF	SVM	XGB	M1	M2	M3	DMOIT	MoGCN
BRCA	mRNA	0.586	0.594	0.612	0.617	**0.658**				—
	MET	0.543	0.556	0.521	0.594	**0.598**				—
	CNV	0.493	0.474	0.452	0.503	**0.557**				—
	mRNA + MET	0.590	0.584	0.579	0.633	0.634	**0.655**	**0.655**	**0.655**	0.541
	mRNA + CNV	0.560	0.600	0.505	0.613	0.648	0.616	0.650	**0.651**	0.534
	MET + CNV	0.535	0.535	0.468	0.590	0.577	0.572	**0.578**	0.574	0.544
	mRNA + MET + CNV	0.562	0.602	0.500	0.626	0.649	0.618	**0.660**	0.619	0.548
HNSC	mRNA	0.619	0.595	0.602	0.597	**0.620**				—
	MET	0.625	0.615	0.603	0.615	**0.631**				—
	CNV	0.593	0.597	0.599	0.616	**0.690**				—
	mRNA + MET	0.634	0.609	0.603	0.623	0.623	0.647	0.630	**0.648**	0.523
	mRNA + CNV	0.616	0.597	0.601	0.612	0.656	0.652	0.667	**0.678**	0.558
	MET + CNV	0.624	0.601	0.603	0.617	0.651	0.660	0.661	**0.672**	0.535
	mRNA + MET + CNV	0.612	0.604	0.603	0.631	**0.669**	0.666	0.662	**0.669**	0.535
LIHC	mRNA	0.629	0.627	0.595	0.633	**0.722**				—
	MET	0.606	0.587	0.552	0.625	**0.679**				—
	CNV	0.588	0.567	0.553	0.578	**0.656**				—
	mRNA + MET	0.609	0.622	0.566	0.632	0.724	0.710	0.721	**0.733**	0.716
	mRNA + CNV	0.605	0.632	0.552	0.631	0.682	0.660	0.697	**0.701**	0.664
	MET + CNV	0.629	0.579	0.549	0.610	0.693	0.643	0.676	**0.694**	0.678
	mRNA + MET + CNV	0.622	0.629	0.550	0.622	0.712	0.682	0.693	**0.727**	0.697
STAD	mRNA	0.542	0.573	0.526	0.558	**0.647**				—
	MET	0.473	0.519	0.461	0.521	**0.572**				—
	CNV	0.523	0.473	0.459	0.511	**0.616**				—
	mRNA + MET	0.491	0.541	0.468	0.566	0.630	0.623	0.637	**0.638**	0.607
	mRNA + CNV	0.506	0.560	0.456	0.544	0.566	0.600	0.601	**0.602**	0.594
	MET + CNV	0.458	0.492	0.460	0.501	**0.592**	0.569	0.578	0.574	0.557
	mRNA + MET + CNV	0.496	0.555	0.457	0.559	0.604	0.594	**0.605**	0.592	0.576

Note: The omics data combinations used include mRNA, MET, CNV, and their integrations. The performance metrics are accuracy and weighted F1 score, averaged over 5-fold cross-validation. The highest values for each metric in each cancer type and omics combination are highlighted in bold.

**TABLE 6:** Performance comparison of various models in the ER classification task using all three types of omics data.

Models	Accuracy	Weighted F1
LR	0.890	0.884
RF	0.917	0.914
SVM	0.878	0.866
Xgboost	0.925	0.923
M1	0.933	0.930
M2	0.933	0.933
M3	**0.945**	0.928
DMOIT	0.937	**0.937**
MoGCN	0.909	0.916

Note: The performance metrics are accuracy and the weighted F1 score, which are averaged over 5-fold cross-validation. The highest values for each metric are highlighted in bold.

**TABLE 7:** Performance of DMOIT across different class ratios in the ER classification task.

ER (+): ER (−)	Accuracy	Weighted F1
1:1	0.927	0.927
2:1	0.933	0.932
3:1	0.914	0.910
199:55	0.937	0.937

### 3.4 Biological findings from DMOIT in estrogen receptor status

To identify potential biomarkers, we employed a permutation importance approach to rank the most important features (Fisher et al., 2019). Specifically, we evaluated the contribution of each feature by shuffling them one at a time during model training, keeping the optimal parameters from the full feature set. We then measured the performance drop in terms of the weighted F1 score to assess how much each feature’s absence impacted the model’s predictive ability (Supplementary Figure S3). We selected features with relatively large decline compared to others, specifically the top 10 from the mRNA dataset, the top 15 from the MET dataset, and the top 4 from the CNV dataset, as shown in Table 8.

**TABLE 8:** Potential biomarkers discovered through the DMOIT in the ER classification task.

	Potential biomarkers
mRNA	PLA2G6, SLC25A38, SLC25A26, FARS2, C2orf15, RBX1, MRFAP1L1, DYNC2LI1, NDUFC1, TRUB2
MET	cg18021992, cg17387069, cg24500294, cg02776659
CNV	MRC2, ATG2A, FRMD8, ARSG, GRB7, HGSNAT, SAC3D1, BATF2, SNX32, OVOL1, RHOD, LRFN4, RCE1, KPNA2

We reviewed previous studies on the top important features from the mRNA and CNV datasets. PLA2G6 and SLC25A26 have been identified as involved in the development of various tumors (Li et al., 2017; Gao et al., 2024), though their link to breast cancer is still underexplored. Elevated expressions of FARS2 and TRUB2 have been noted in breast cancer tissues, and C2orf15 expression significantly correlates with breast cancer prognosis, although their link to ER status needs further investigation (Sung et al., 2022; Tau et al., 2024; Mi et al., 2024). SLC25A38 is known to upregulate ER expression, while Rbx1 is essential for the degradation of ERα protein, playing a critical role in estrogen signaling (Lu et al., 2023; Marconett et al., 2010). NDUFC1, important in mitochondrial metabolism, has been found to be more critical in ER + breast cancer cells, suggesting a metabolic vulnerability in this subtype (Tau et al., 2024). MRFAP1L1 and DYNC2LI1 show promise as potential biomarkers, although no studies have yet indicated an association with breast cancer. For CNV biomarkers, MRC2 amplification and copy number gain in basal-like breast cancer may be linked to tumorigenesis and progression. Since basal-like tumors are typically ER-, this may suggest a potential connection (Wienke et al., 2007). ATG2A exhibits mutations in breast cancer, FRMD8 plays a tumor-suppressive role in breast cancer progression, ARSG is negatively correlated with positive prognosis, and differentially expressed genes upregulated by SAC3D1 are involved in regulating the cell cycle pathways in breast cancer cells. However, no research has yet examined the impact of copy number variations of these genes on ER status (Wu et al., 2021; Wu et al., 2024; Alkhateeb et al., 2020; Liu et al., 2020). GRB7 is overexpressed in breast cancer cell lines, showing a strong correlation between mRNA levels and copy number status. It is essential for the invasion and survival of triple-negative breast cancer cells (Staaf et al., 2010; Giricz et al., 2012). OVOL1 is highly expressed in ER + breast cancer (Fan et al., 2022), while RHOD has a causal role specifically in ER + breast cancer (Kazmi et al., 2022). SNX32 leads to frequent loss-of-function mutations in breast cancer patients (Li et al., 2018). The novel Ras membrane-bound regulator of Ras, Rce1, suggests a promising strategy for targeting Ras in breast cancer (Hanker and Der, 2010), and KPNA2 overexpression significantly enhances the invasion and migration capabilities of breast cancer cells (Han and Wang, 2020). The role of LRFN4, BATF2, and HGSNAT in breast cancer remains unexplored. These findings suggest that DMOIT successfully identifies potential biomarkers, enhancing its value in breast cancer studies.

Furthermore, we assessed the joint effects of multiple omics biomarkers using multiple linear regression. Specifically, we analyzed the direction of the coefficient for the biomarker $X_{1}$ in the model $Y=a_{1}X_{1}$ when only a single omics biomarker was present, and compared it to the direction of the coefficient for $X_{1}$ in the model Y = $a_{1}X_{1}$ + ${a_{2}X}_{2}$ + ${a_{3}X}_{3}$ , which included three omics biomarkers. We explored all 560 possible combinations, from 10 mRNA, 14 CNV, and 4 MET biomarkers. When $X_{1\;}$ was an mRNA biomarker, the inclusion of $X_{2\;}$ and $X_{3}$ did not change the direction of $X_{1}$ ’s coefficient. However, when $X_{1}$ was an MET biomarker, 78 out of 560 combinations resulted in a change. Similarly, when CNV served as $X_{1}$ , 63 out of 560 combinations caused a shift in the direction of $X_{1}$ . These findings suggest that the joint effects of CNV and MET on mRNA may be relatively weak. In contrast, the joint effects of MET and mRNA on CNV are stronger, while the strongest joint effects are observed between CNV and mRNA on MET.

## 4 Discussion

In this study, we propose DMOIT, a denoised multi-head self-attention-based multi-omics integration framework that considers both intra- and inter-omics interactions. DMOIT introduces the GAIN module for imputation, the RFS module for feature selection, and multi-head self-attention layers for feature extraction. We investigated the effectiveness of each component in DMOIT, finding that the GAIN module can be generalized well across different omics types, effectively reducing noise from inappropriate imputation methods. Additionally, the RFS module successfully identifies stable and denoised features, reducing redundancy and noise, which enhances the data quality and improves downstream analyses performance. Furthermore, our designed MHSA mechanism-based integration model, outperforms traditional machine learning models and other MHSA-based methods across diverse cancer types and varying omics combinations.

However, our study has several limitations that warrant consideration. First, deep learning-based methods operate as black boxes and lack interpretability (Toussaint et al., 2024). This characteristic makes it challenging to understand the underlying decision-making processes and limits the insights that can be drawn from the model. As a result, the practical application value of such deep learning models in clinical settings may be restricted. To address this issue, we propose that researchers in related fields apply more knowledge from the realm of explainable AI to enhance model interpretability and provide clinicians with visualization tools that can aid in understanding model predictions, thereby increasing the feasibility of clinical applications. Second, this study focuses on optimizing integration procedures based on high-dimensional data characteristics without incorporating biological knowledge. By exclusively prioritizing data-driven optimization, the framework risks missing out on valuable biological insights that could enhance both its predictive power and interpretability. Future studies should integrate biological insights into feature selection and extraction processes, such as incorporating pathway information into the attention mechanisms to enhance model interpretability and provide more meaningful insights into the biological mechanisms underlying the data (Crawford and Greene, 2020). Furthermore, our experimental results showed that DMOIT achieved optimal performance across all omics combinations in the LIHC and HNSC datasets, but not for BRCA and STAD datasets. This indicates that the model’s effectiveness may vary with specific omics combinations and cancer types. Future studies should explore different architecture configurations and assess how various omics combinations influence interaction strength. Investing these aspects will help refine the model to better accommodate diverse mics data and improve its overall performance. Additionally, exploring the model’s adaptability to other omics types and evaluating its performance in different clinical settings could provide further validation and improvements.

In conclusion, our proposed approach effectively integrates multi-omics data by addressing noise reduction and feature stability while considering both intra- and inter-omics interactions. It demonstrates superior performance and stability, making it a promising tool for multi-omics research.

## Data Availability

The data presented in the study are deposited in the UCSC Xena repository, accession number GDC TCGA Breast Cancer (BRCA), GDC TCGA Head and Neck Cancer (HNSC), GDC TCGA Liver Cancer (LIHC) and GDC TCGA Stomach Cancer (STAD).
